# Anomalous systemic arterial supply to the basal segment of the lung with giant aberrant artery: a case report

**DOI:** 10.1186/s40792-020-01063-w

**Published:** 2020-11-12

**Authors:** Takahiro Utsumi, Haruaki Hino, Shintaro Kuwauchi, Nobuya Zempo, Kaori Ishida, Natsumi Maru, Hiroshi Matsui, Yohei Taniguchi, Tomohito Saito, Koji Tsuta, Tomohiro Murakawa

**Affiliations:** 1 Department of Thoracic Surgery, Kansai Medical University Hospital, 2-3-1 Shin-machi, Hirakata, Osaka 573-1191 Japan; 2 Department of Cardiovascular Surgery, Kansai Medical University Hospital, Osaka, Japan; 3 Department of Pathology and Laboratory Medicine, Kansai Medical University Hospital, Osaka, Japan

**Keywords:** Anomalous systemic arterial supply to the basal segment of the lung, Giant aberrant artery, Staged surgical therapy

## Abstract

**Background:**

Anomalous systemic arterial supply to the basal segment of the lung (ABLL) is a relatively rare congenital anomaly characterized by aberrant systemic arterial blood flow to the basal segment of the lung. We experienced a rare presentation of ABLL, in which a giant aberrant artery with the same dimensions as that of the descending aorta flowed from the celiac artery to left lower lobe.

**Case presentation:**

An otherwise healthy 42-year-old man was referred to our department due to an abnormal chest X-ray. Enhanced computed tomography revealed a huge and winding aberrant artery with mural thrombus originating from the celiac artery and perfusing into the left lower lobe. We diagnosed giant ABLL and considered possible concomitant pulmonary arteriovenous fistula. The diameter of the aberrant artery was > 30 mm and high-pressure flow was assumed; therefore, we performed staged resection of the left lower lobectomy including division of the aberrant artery at the pulmonary ligament and subsequent embolization of the remnant arterial flow uneventfully. Pathologically, the aberrant artery was abundant with elastic fibers, and dissections of the tunica media and mural thrombus were observed; however, arteriovenous fistula was not confirmed. At 6 postoperative months, enhanced computed tomography showed the aberrant artery to be completely occluded without any symptoms.

**Conclusions:**

We present a case of ABLL that was successfully managed by surgical resection of the left lower lobe with most of the giant aberrant artery and subsequent embolization of the remnant portion. Our study demonstrates that a staged surgical therapy is an acceptable approach for ABLL in case of complication with a giant aberrant artery.

## Background

Anomalous systemic arterial supply to the basal segment of the lung (ABLL) is a relatively rare congenital anomaly characterized by aberrant systemic arterial blood flow to the basal segment of the normal lung drained to the pulmonary vein; however, the position of the bronchus is at the normal branch. The condition requires treatment due to the potential risk of complications such as pulmonary hypertension, heart failure, hemoptysis and rupture of the aberrant artery [1, 2]. We report an extremely rare presentation of ABLL with a giant aberrant artery as large as the descending aorta that arose from the celiac artery to left lower lobe.

## Case presentation

**Fig. 1:**
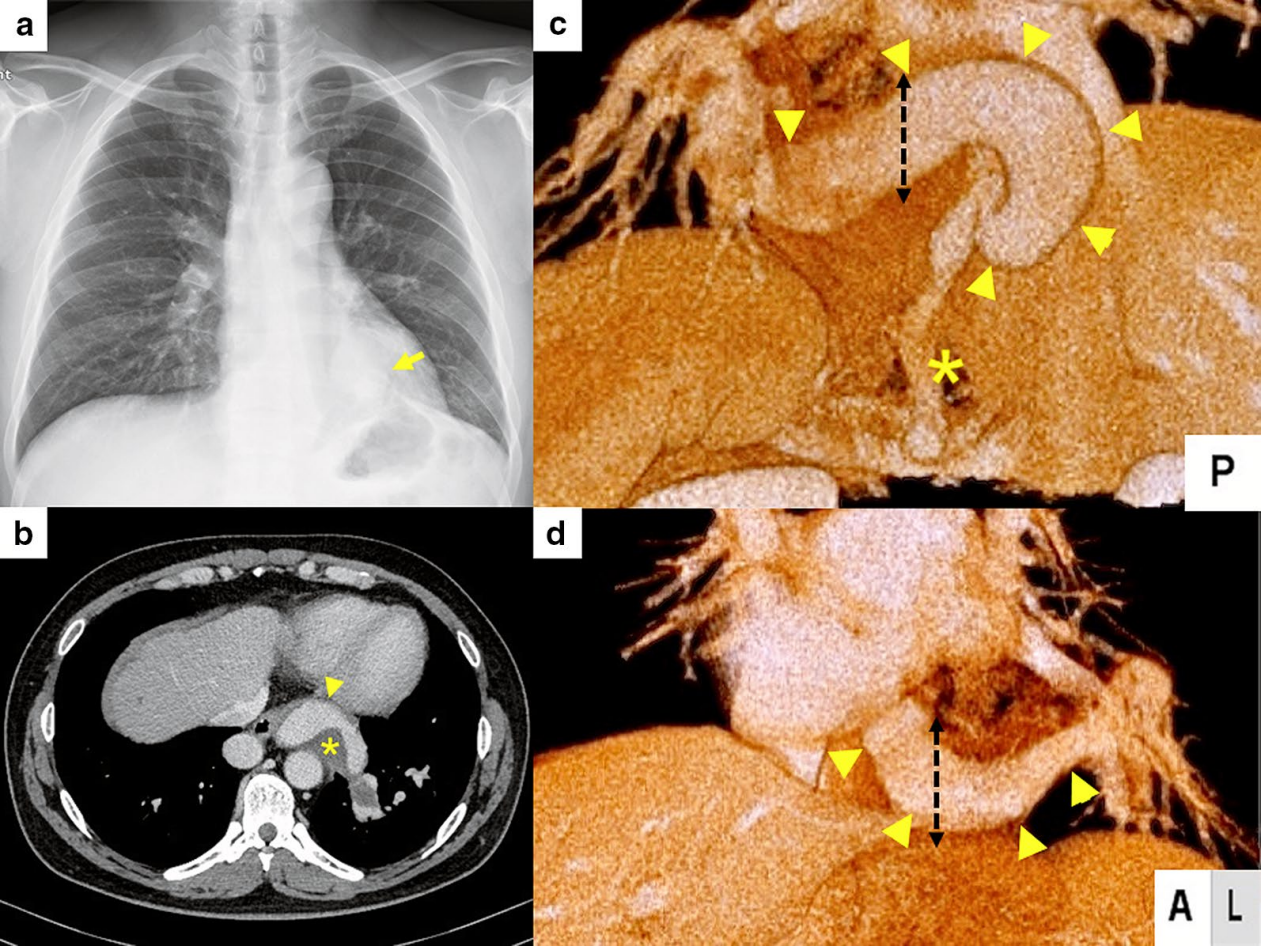
Preoperative chest X-ray and computed tomography images. **a** Chest X-ray image showing abnormal consolidation in the left lower lung field (yellow arrow). **b** Enhanced computed tomography revealed the presence of an aberrant artery with a maximum diameter of 33 mm (yellow arrowheads) with a thrombosed lumen (asterisk). **c** Three-dimensional computed tomography from posterior view revealed an aneurysmal aberrant artery (yellow arrowheads) arising from the celiac artery. We resected the aberrant artery with pulmonary ligament (black dotted line). After surgery, we performed coil embolization on the residual aberrant artery (asterisk). **d** Three-dimensional computed tomography from anterior view showed that the aneurysmal aberrant artery (yellow arrowheads) arose from the celiac artery and was flowing into the left lower lobe along the pulmonary ligament forming an aneurysm. It was divided along with the pulmonary ligament (black dotted line)

A healthy 42-year-old man was found to have an abnormal chest shadow during an annual medical examination and was referred to our hospital (Fig. 1a). Enhanced computed tomography (CT) revealed a thrombosed aberrant artery with a maximum diameter of 33 mm arising from the celiac artery and flowing to the left lower lobe through the pulmonary ligament, draining into the left inferior pulmonary vein (Fig. 1b, c). Based on the above findings, we diagnosed ABLL with giant aberrant artery. In addition, we considered the possibility of concomitant pulmonary arteriovenous fistula because the aberrant artery was too large for a single disease of ABLL, and aneurysmal aberrant artery was assumed to be an effect of pulmonary arteriovenous fistula. Therefore, we performed surgical therapy to shut down abnormal blood flow and reduce the risk of rupture [3]. In addition, according to a prior report, the stump of an aberrant artery may form an aneurysm; therefore, we planned to perform left lower lobectomy with subsequent coil embolization of the remnant aberrant artery when the blood supply was confirmed postoperatively [4].

**Fig. 2:**
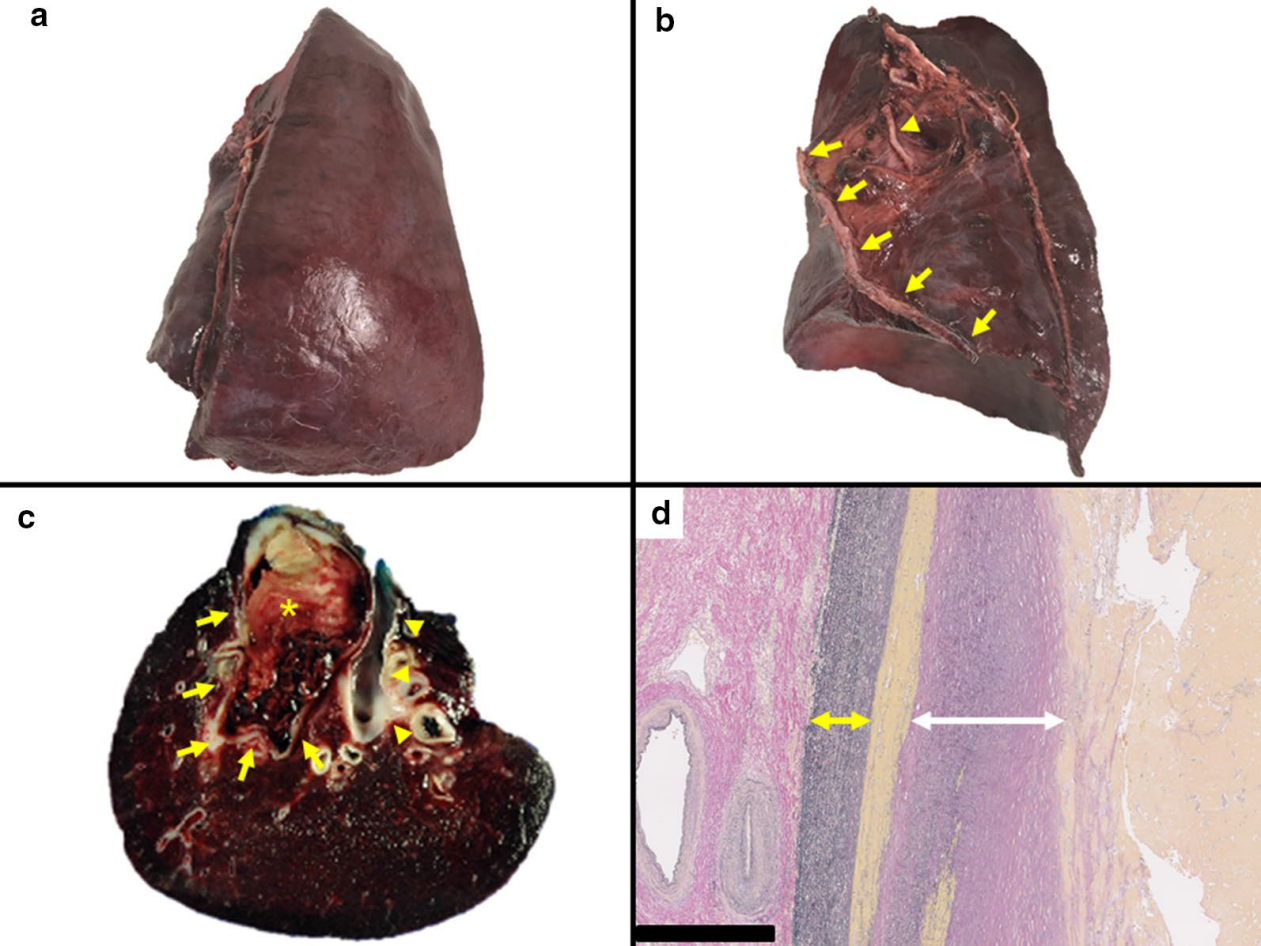
Pathological findings. **a** Photograph of the resected left lower lobe showing congestion (frontal view). **b** Photograph showing the aberrant artery including pulmonary ligament (yellow arrows), which was large and thick compared with the left inferior pulmonary vein (yellow arrowhead) (hilar view). **c** Photograph of the aberrant artery (yellow arrows), which was larger than the left inferior pulmonary vein (yellow arrowheads), and the thrombus was filled in the lumen (asterisk). **d** Elastica van Gieson-stained sections showing the aberrant artery with thick elastic lamina and dissected between the tunica media (yellow double-headed arrow) and intima (white double-headed arrow). Scale bar = 500 µm

**Fig. 3:**
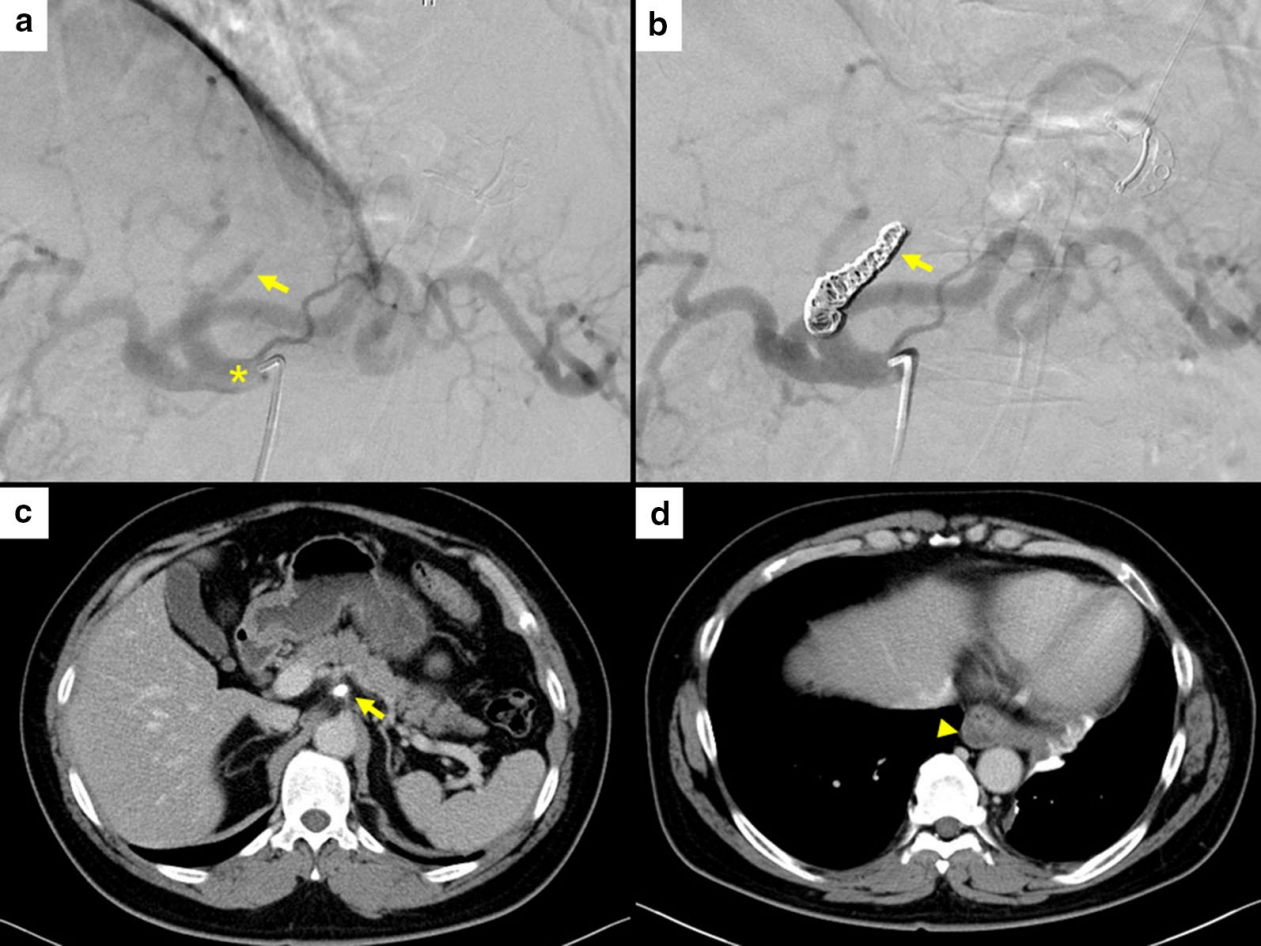
Interventional radiology and postoperative computed tomography images. **a** Celiac angiography (asterisk) showing slight residual blood flow in the aberrant artery (yellow arrow). **b** After coil embolization, the aberrant artery was found to be completely occluded (yellow arrow). **c** Computed tomography image showing occlusion of the aberrant artery at the celiac artery 6 months after surgery (yellow arrow). **d** Computed tomography image showing the shrunken remnant aberrant artery 6 months after surgery (yellow arrowhead)

We performed the left lower lobectomy with aberrant artery excision under thoracotomy (20 cm antero-axial incision). The giant aberrant artery was located along the pulmonary ligament. The left inferior pulmonary vein was resected firstly to prevent thromboembolism given the possible pulmonary arteriovenous fistula; thereafter, the left lower lobe gradually became congested before the aberrant artery shut down. Although subsequent procedures were complicated by the congested lung, the aberrant artery was divided with the pulmonary ligament using an endoscopic stapler (Echelon FlexTM GST System with 45-mm black reloads; three cartridges [Ethicon Endo-Surgery Inc., Cincinnati, OH, USA]) under decompression using a clamp at the proximal portion of the aneurysmal aberrant artery. Although the aberrant artery had aneurysms, the aortic wall revealed enough elasticity and firmness; therefore, we resected the aneurysmal aberrant artery with pulmonary ligament using auto-suture device and the stump was reinforced using Teflon pledgets [5] (Fig. 2a, b). The operation time was 210 min with a total blood loss of 1586 mL without blood transfusion. Most of the bleeding originated from a damaged pleural of the congested left lower lobe itself, from the stapled cut ends of the pulmonary artery, and vein in resected lung, and not from the central side of the aberrant artery. Preoperative CT showed that most parts of the aberrant artery lumen were thrombosed; we expected central thrombotic occlusion of the aberrant artery after resection of the peripheral aberrant artery. However, we confirmed by enhanced CT that the blood supply to the remnant aberrant artery remained; therefore, we planned to perform coil embolization. At 18 days after the first operation, angiography revealed slight residual blood flow in the aberrant artery toward the surgical stump through the diaphragm, which was completely embolized using a fibered interlocking detachable coil (Boston Scientific, Natick, MA, USA) (Fig. 3a, b). The two surgeries and postoperative course were uneventful.

Pathologically, the tunica media of the aberrant artery contained abundant elastic fibers, and dissection of the tunica media and mural thrombus were observed; however, arteriovenous fistula was not confirmed (Fig. 2c, d). At 6 postoperative months, the patient did not show any symptoms, and enhanced CT findings confirmed the aberrant artery to be completely occluded with no aneurysms (Fig. 3c, d).

## Discussion

ABLL has previously been classified as Pryce’s type I pulmonary sequestration, but has become recognized as a pulmonary vessel malformation that is different from pulmonary sequestration [6]. Most previously reported cases describe aberrant arteries arising from the descending aorta; reports of ABLL in which the aberrant artery arises from the celiac artery, as in the present case, are relatively rare (6%) [7]. The median diameter of the aberrant artery has been reported to be 10.0 mm (range 4.0–20.0 mm); the aberrant artery in our case is the larger than past reports [7]. This meant that a two-staged approach was required in the present case, in contrast to the methods reported in other studies.

Surgical resection of the aberrant artery and ipsilateral lobectomy are generally performed for patients with ABLL, and embolization is indicated for inoperable cases [8]. Recently, certain studies have reported the division of blood flow from an aberrant artery without pulmonary resection with an uneventful postoperative course [9, 10]. In the present case, given the large diameter of the aberrant artery, we considered that preoperative reduction of blood flow of the aberrant artery by embolization was a safe approach to prevent unexpected intraoperative bleeding. However, coil embolization is not recommended in cases where the inner vessel diameter is 10 mm or greater because of the risk of incomplete occlusion and coil migration [11]. Even though embolization for a narrow segment of the aberrant artery was an option, we prioritized shutting down the abnormal blood flow of the pulmonary arteriovenous fistula; therefore, we first performed surgical resection of the aberrant artery and left lower lobectomy prior to coil embolization for the remnant aberrant artery as a staged procedure. There is one report of successful embolization of an aberrant artery in an operable patient [12]; however, no other case studies have performed combined operative procedures with resection of the aberrant artery and subsequent coil embolization for remnant flow, partly because the aberrant artery is too large and partly because there might be a possibility of concomitant pulmonary arteriovenous fistula. Although we performed an unprecedented staged operation, it might be a feasible procedure in such cases, and as a result, the postoperative course was uneventful.

With regard to surgical procedures, it has been reported that the resection of aberrant arteries using an endoscopic stapler is a safe procedure, which we performed the same way [5]. Before surgery, we were concerned about using an endoscopic stapler for the aneurysmal aberrant artery; however, intraoperatively, we confirmed that the artery wall was thick and firm; therefore, we resected it using an endostapler. Furthermore, the diameter of the patient’s aberrant artery was the same as the descending aorta, which was larger than the previously reported diameters. Thus, we reinforced the stump using Teflon pledgets without any complications [7]. During the surgery, a resection of the left lower pulmonary vein was performed first because the presence of preoperative pulmonary arteriovenous fistula was possible; however, as a result of rapid congestion of the left lower lobe, it was considered that the resection of the aberrant artery prior to pulmonary vein might be desirable to prevent ipsilateral pulmonary congestion. The good outcome of this case indicates that the optimal surgical strategy for variable ABLL should be considered and decided depending on each case.

Considering the histopathological features of the aberrant artery, the presence of an elastic artery with dissection of the tunica media might be complicated when an aneurysm forms with high blood flow, which is the same as our case. Indeed, there are several reports of aneurysms of aberrant arteries leading to fatal ruptures [2, 3]. Giant aberrant arteries like that of the present case carry the risk of dissection and rupture; therefore, appropriate treatment must be carried out in a timely manner following diagnosis.

## Conclusions

We present a case of ABLL which was successfully managed by surgical resection of the left lower lobe with most of the giant aberrant artery and subsequent embolization of the remnant portion. Our study demonstrates that a staged surgical therapy can be an acceptable procedure for ABLL in cases with a giant aberrant artery.

## Data Availability

All datasets presented in the main paper are available whenever possible.

## Abbreviations

ABLL: Anomalous systemic arterial supply to the basal segment of the lung
CT: Computed tomography
